# Patient safety considerations using dose-response models in external radiation therapy of head-&-neck and breast cancers: clinical-dosimetric predictors of radiation-induced hypothyroidism

**Published:** 2019-08

**Authors:** Aysan Mohammad-Namdar, Homayoun Sadeghi-Bazargani, Amir Hooman Kazemi-Motlagh, Asghar Mesbahi, Mohammad Mohammadzadeh

**Affiliations:** ^*a*^Immunology Research Center. Tabriz University of Medical Sciences, Tabriz, Iran.; ^*b*^Road and Traffic Injury Research Center, Department of Statistics and Epidemiology, Faculty of Health, Tabriz University of Medical Sciences, Tabriz, Iran.; ^*c*^Beijing University of Chinese Medicine, Beijing, china.; ^*d*^Molecular Medicine Research Center, Tabriz University of Medical Sciences, Tabriz, Iran.; ^*e*^Molecular Medicine Research Center, Institute of Biomedicine, Tabriz University of Medical Sciences, Tabriz, Iran

## Abstract

**Background:**

The effectiveness of the six mathematical models was evaluated for prediction of radiation-induced hypothyroidism (RHT) in patients with head-and-neck cancer (HNC) and breast cancer (BC).

**Methods:**

Clinical and dose-volume data of 62 patients treated with three-dimensional conformal radiation therapy (3DCRT) for HNC and BC were prospectively analyzed. Thyroid function assessment was evaluated by the level of thyroid hormones from patient serum sample. Cox semi-parametric regression models were used to predict the hazard of RHT. Model performance and model ranking was evaluated through the area under the receiver operating characteristic curve (AUC) and Akaike's information criterion (AIC), respectively.

**Results:**

17 of 62 patients (median 53 years) developed RHT at a median follow-up of 11.4 months after radiotherapy. Kaplan-Meier curves showed a decrease in RHT hazard with thyroid mean dose less than 31 Gy when applied on the whole dataset. Simple and multiple analysis on the whole dataset revealed that RHT hazard was higher for smaller thyroid volumes. For patients with HNC, Vthyroid (thyroid gland volume) and Dmean (thyroid gland mean dose) were found to be influencing factors in prediction of RHT. Based on the AUC, Boomsma’s model and generalized equivalent-uniform-dose (EUD) model outperformed on the whole dataset. The ROC comparison showed no significant difference in the prediction capability of the six models on the whole dataset. Boomsma and gEUD models were ranked as the best models based on the AIC value.

**Conclusions:**

In this study, Vthyroid and Dmean were predominant variables for prediction of RHT. Boomsma and gEUD models provided a good prediction power for RHT hazard on the whole and HNC datasets. In conclusion, using these models in the clinic will fulfil the safety of patients against the complication probability following radiation therapy as high as possible.

**Keywords:**

Dose-response models, Radiation therapy, Patient safety

